# Supplementary material for: Identification of Freezing-Responsive microRNAs and Their Targets in Chinese Jujube by Small RNA and Degradome Sequencing
Source: Int J Mol Sci. 2026 Sep 4;27(17):7912. doi: 10.3390/ijms27177912 (PMC13566890; doi:10.3390/ijms27177912)
Supplement: Supplementary file 1 [file ijms-27-07912-s001.zip › Supplementary Material S2/novel_miR_205.pdf]

Provisional ID	:	novel_miR_205
Score total	:	333.3
Score for star read(s)	:	3.9
Score for read counts	:	331.5
Score for mfe	:	0.1
Score for randfold	:	-2.2
Score for cons. seed	:	
Total read count	:	662
Mature read count	:	473
Loop read count	:	108
Star read count	:	81

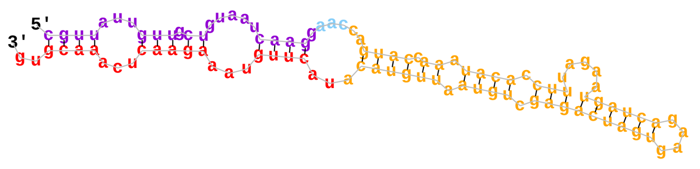

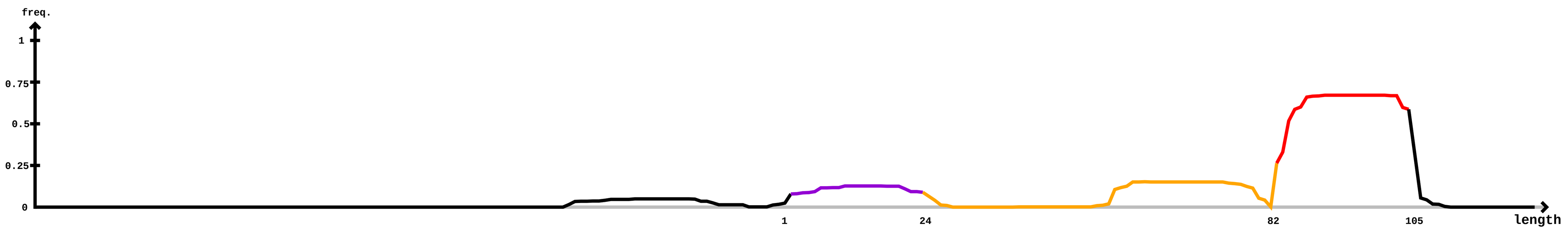

Star Mature

```
5'- aaaacaaauauaaaauugauucaaccccaagccauuugaauguaguggauaacaguggagcccaagaauuaaugaguauuugaaucguaggagcuaguaaucgacgauaugcucauauuggugacguuauuguugcuguaaucaaggaaccaguaccaaauacaccuuuagaaugaucagaagugaucagagcuguaauuguacauacuuguaaagaacucaaacgugagaacgauaugaucauacgaca -3' obs
    aaaacaaauauaaaauugauucaaccccaagccauuugaauguaguggauaacaguggagcccaagaauuaaugaguauuugaaucguaggagcuaguaaucgacgauaugcucauauuggugacguuauuguugcuguaaucaaggaaccaguaccaaauacaccuuuagaaugaucagaagugaucagagcuguaauuguacauacuuguaaagaacucaaacgugagaacgauaugaucauacgaca     exp
    ....(((((((...((((((((...((((..(((((.......))))).......))).).....))))))))..)))))))..((((..((........)).))))((((.((((((((...(((((...(((.((.....((((......((((.((.((((.(((.....((((((....))))))))).)))).))))))...))))...)))))...)))))....)))))))).)))).....
```

read	reads	mm	sample
uaggagcuaguaaucgacgaua	2	0	LT-1
uaggagcuaguaaucgacgaua	2	0	JCK-2
uaggagcuaguaaucgacgauaug	1	0	JCK-2
uaggagcuaguaaucgacgauaugc	6	0	LCK-3
aggagcuaguaaucgacgau	1	0	LT-3
aggagcuaguaaucgacgaua	3	0	LT-2
aggagcuaguaaucgacgaua	1	0	JCK-3
aggagcuaguaaucgacgaua	1	0	JCK-1
aggagcuaguaaucAacgauaug	1	1	JCK-3
aggagcuaguaaucgacgauaug	1	0	LT-1
aggagcuaguaaucAacgauaug	1	1	JCK-2
aggagcuaguaaucgacgauauU	1	1	LT-2
aggagcuaguaaucgacgauaug	1	0	JCK-3
aggagcuaguaaucgacgauaugc	2	0	LT-2
ggagcuaguaaucgacgauaugc	1	0	JCK-2
agcuaguaaucgacgauaug	1	0	LT-3
cuaguaaucgacgauaugcucaua	2	0	JCK-3
cuaguaaucgacgauaugcucaua	1	0	JCK-2
uaguaaucgacgauaugcucaua	3	0	LT-2
uaguaaucgacgauaugcucaua	1	0	LT-1
aaucgacgauaugcucaua	1	0	JCK-2
aaucgacgauaugcucaua	1	0	JCK-3
ucauauuggugacguuauuguugcugua	1	0	LT-1
ugacguuauuguugcuguaauca	3	0	JCK-1
ugacguuauuguugcuguaauca	2	0	JCK-2
ugacguuauuguugcuguaauca	3	0	JCK-3
gacguuauuguugcuguaaucaaggaC	1	1	LT-1
gacguuauuguugcuguaaucaaggaCc	1	1	LT-1
gacguuauuguugcuguaaucaaggaac	1	0	LT-3
acguuauuguugcuguaauca	1	0	LT-3
acguuauuguugcuguaaucaag	2	0	LT-3
acguuauuguugcuguaaucaagg	1	0	JCK-1
acguuauuguugcuguaaucaagg	1	0	JCK-2

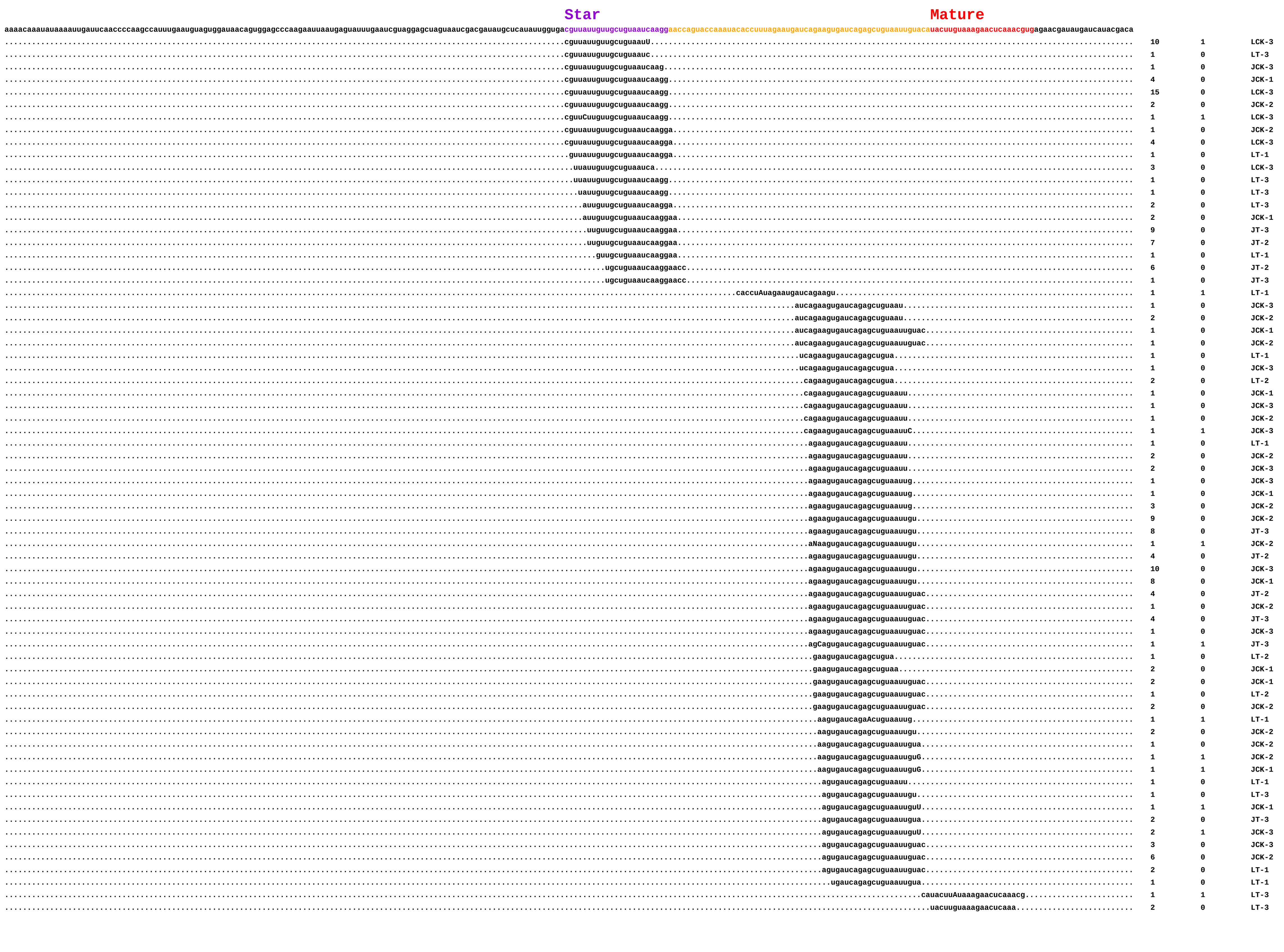
Star
Mature

Star | Mature

aaaacaaauauaaaauugauucaaccccaagccauuugaauguaguggauaacaguggagcccaagaauuaaugaguauuugaaucguaggagcuaguaaucgacgauaugcucauauuggugacguuauuguugcuguaaucaaggaaccaguaccaaauacaccuuuagaaugaucagaagugaucagagcuguaauuguacauacuuguaaagaacucaaacgugagaacgauaugaucauacgaca

uacuuguaaagaacucaaacg	24	0	LCK-2
uacuuguaaagaacucaaacg	23	0	LCK-1
uacuuguaaagaacucaaacgu	2	0	LT-1
uacuuguaaagaacucaaacgu	1	0	LT-3
uacuuguaaagaacucaaacgug	4	0	JCK-3
uacuuguaaagaacucaGacgug	2	1	JCK-1
uacuuguaaagaacucaaacgug	9	0	JCK-1
uacuuguaaagaacucaaacgug	8	0	JT-2
uacuuguaaaUaacucaaacgug	1	1	LT-3
uCcuuguaaagaacucaaacgug	1	1	LCK-3
uacuuguaaagaacucaaacgug	6	0	JT-3
uacuuguaaagaacucaaacgug	6	0	LT-1
uacuuguaaagaacucaaacgug	18	0	LT-2
uacuuguaaagaacucaaacgug	11	0	JCK-2
uacuuguaaagaacucaaacgug	11	0	LT-3
uacuuguaaagaacucaaacgug	40	0	LCK-3
uacuuguaaagaacucaaacguga	4	0	JT-3
uacuuguaaagaacucaaacguga	4	0	JT-2
uacuuguaaagaacucaaacguga	2	0	JCK-2
uacuuguaaagaacucaaacguga	1	0	JCK-3
uacuuguaaagaacucaaacgugag	2	0	JCK-3
uacuuguaaagaacucaaacgugag	3	0	JCK-2
uacuuguaaagaacucaaacgugag	1	0	JCK-1
acuuguaaagaacucaaacgu	1	0	JCK-2
acuuguaaagaacucaaacgu	2	0	JCK-3
acuuguaaagaacucaaacgug	4	0	JCK-1
acuuguaaagaacucaaacgug	6	0	JCK-3
acuuguaaagaacucaaacgug	1	0	JT-3
acuuguaaagaacucaaacgug	2	0	LT-1
acuuguaaagaacucaaacgug	7	0	LCK-3
acuuguaaagaacucaaacgug	2	0	LT-3
acuuguaaagaacucaaacgug	6	0	JCK-2
acuuguaaagaacucaaacgug	11	0	LT-2
acuuguaaagaacucaaacgugag	2	0	LT-2
acuuguaaagaacucaaacgugaga	3	0	LT-2
cuuguaaagaacucaaacg	1	0	LT-1
cuuguaaagaacucaaacg	1	0	LT-3
cuuguaaagaacucaaacgu	1	0	JCK-3
cuuguaaagaacucaaacgu	1	0	JCK-1
cuuguaaagaCcucaaacgug	1	1	LT-1
cuuguaaagaacucaaacgug	8	0	JCK-3
cuuguaaagaacucaaacgug	6	0	LT-3
cuuguaaagaacucaaacgug	5	0	JT-2
cuuguaaagaacucaaacgug	4	0	LCK-3
cuuguaaagaacucaaacgug	21	0	LCK-2
cuuguaaagaacucaaacgug	4	0	JCK-1
cuuguaaagaacNcaaacgug	1	1	LCK-1
cuuguaaagaacucaaacgug	10	0	JCK-2
cuuguaaagaCcucaaacgug	1	1	LCK-3
cuuguaaagaacucaaacgug	38	0	LCK-1
cuuguaaagaacucaaacgug	7	0	LT-2
cuuguaaagaacucaaacgug	5	0	JT-3
cuuguaaagaacucaaacguga	1	0	JCK-2
cuuguaaagaacucaaacgugU	1	1	JCK-1
cuuguaaagaacucaaacgugU	2	1	JCK-2
cuuguaaagaacucaaacgugU	1	1	JCK-3
cuuguaaagaacucaaacguga	2	0	JCK-3
cuuguaaagaacucaaacguga	2	0	JCK-1
cuuguaaagaacucaaacgugaga	5	0	LCK-3
cuuguaaagaacucaaacgugagaac	2	0	LCK-3
cuuguaaagaacucaaacgugagaac	1	0	JCK-1
cuuguaaagaacucaaacgugagaac	1	0	JCK-2
uuguaaagaacucaaacgug	2	0	LT-1
uuguaaagaacucaaacgug	11	0	JT-1
uuguaaagaacucaaacgug	2	0	JCK-3
uuguaaagaacucaaacgug	4	0	LCK-3
uuguaaagaacucaaacgug	3	0	LT-3
uuguaaagaacucaaacgug	11	0	LT-2
uuguaaagaacucaaacguga	1	0	JCK-2
uuguaaagaacucaaacguga	2	0	JCK-1

```
                                                                                                                            Star                                                                                   Mature
aaaacaaauauaaaauugauucaaccccaagccauuugaauguaguggauaacaguggagcccaagaauuaaugaguauuugaaucguaggagcuaguaaucgacgauaugcucauauuggugacguuauuguugcuguaaucaaggaaccaguaccaaauacaccuuuagaaugaucagaagugaucagagcuguaauuguacauacuuguaaagaacucaaacgugagaacgauaugaucauacgaca
................................................................................................................................................................................................................uuguaaagaacucaaacgugU.....................   4   1   LT-2
................................................................................................................................................................................................................uuguaaagaacucaaacgugU.....................   2   1   JCK-1
................................................................................................................................................................................................................uuguaaagaacucaaacgugaga...................   3   0   LT-2
................................................................................................................................................................................................................uuguaaagaacucaaacgugagaac.................   4   0   LCK-3
.................................................................................................................................................................................................................uguaaagaacucaaacgug......................   9   0   JT-1
.................................................................................................................................................................................................................uguaaagaacucaaacgug......................   1   0   JCK-2
..................................................................................................................................................................................................................guaaagaacucaaacgug......................   3   0   LT-1
..................................................................................................................................................................................................................Auaaagaacucaaacgug......................   1   1   JCK-2
..................................................................................................................................................................................................................guaaagaacucaaacgug......................   4   0   LCK-3
..................................................................................................................................................................................................................guaaagaacucaaacgug......................   14  0   JCK-2
..................................................................................................................................................................................................................guaaagaacucaaacgug......................   14  0   JT-1
..................................................................................................................................................................................................................guaaagaacucaaacgugU.....................   2   1   LT-2
..................................................................................................................................................................................................................guaaagaacucaaacgugaga...................   1   0   LT-3
..................................................................................................................................................................................................................guaaagaacucaaacgugagaacg................   1   0   JCK-1
..................................................................................................................................................................................................................guaaagaacucaaacgugagaacg................   2   0   JCK-2
...................................................................................................................................................................................................................uaaagaacucaaacgugaga...................   4   0   LCK-3
....................................................................................................................................................................................................................aaagaacucaaacgugagaac.................   1   0   LT-1
.....................................................................................................................................................................................................................aagaacucaaacgugaUa...................   2   1   LT-2
.....................................................................................................................................................................................................................aagaacucaaacgugagaa..................   1   0   LT-1
```
